# Greater muscle volume and muscle fat infiltrate in the deep cervical spine extensor muscles (multifidus with semispinalis cervicis) in individuals with chronic idiopathic neck pain compared to age and sex-matched asymptomatic controls: a cross-sectional study

**DOI:** 10.1186/s12891-022-05924-3

**Published:** 2022-11-10

**Authors:** Suzanne J Snodgrass, Peter Stanwell, Kenneth A. Weber, Samala Shepherd, Olivia Kennedy, Hannah J Thompson, James M Elliott

**Affiliations:** 1 School of Health Sciences, The University of Newcastle, University Drive, Callaghan, Australia; 2 Hunter Medical Research Institute, New Lambton Heights, Australia; 3 Department of Anesthesiology, Perioperative and Pain Medicine, Stanford University School of Medicine, Palo Alto, CA USA; 4 The University of Sydney, Faculty of Medicine and Health & The Northern Sydney Local Health District - The Kolling Institute, Level 13, NSW St Leonards, Australia; 5 Feinberg School of Medicine, Northwestern University, Chicago, IL USA

**Keywords:** Neck muscles, Muscle, skeletal, Neck pain, Musculoskeletal pain, Chronic pain

## Abstract

Muscle size and composition (muscle volume and muscle fat infiltrate [MFI]) may provide insight into possible mechanisms underpinning chronic idiopathic neck pain, a common condition with no definitive underlying pathology. In individuals with chronic idiopathic neck pain > 3 months and age- and sex-matched asymptomatic controls, muscle volumes of levator scapulae, multifidus including semispinalis cervicis (MFSS), semispinalis capitis, splenius capitis including splenius cervicis (SCSC), sternocleidomastoid and longus colli from C3 through T1 were quantified from magnetic resonance imaging. Between-group differences were determined using linear mixed models, accounting for side (left or right), muscle, spinal level, sex, age, and body mass index (BMI). Individuals with pain had greater muscle volume (mean difference 76.8mm^3^; 95% CI 26.6–127.0; *p* = .003) and MFI (2.3%; 0.2–4.5; *p* = .034) of the MFSS compared to matched controls with no differences in relative volume, accounting for factors associated with the outcomes: muscle, spinal level, side (left had smaller volume, relative volume and MFI than right), sex (females had less volume and relative volume than males), age (older age associated with less relative volume and greater MFI), and BMI (higher BMI associated with greater muscle volume and MFI). Greater MFI in individuals with chronic idiopathic neck pain suggests a possible underlying mechanism contributing to neck pain. Perspective: These findings suggest MFI in the MFSS may be radiologic sign, potentially identifying patients with a less favourable prognosis. Future studies are needed to confirm this finding and determine if MFI is a contributor to the development or persistence of neck pain, or consequence of neck pain.

**Supplementary Information:**

The online version contains supplementary material available at 10.1186/s12891-022-05924-3.

## Background

Neck pain is the 4th greatest contributor to years lived with disability globally [1], and this burden is likely underestimated [2]. Research into prevention and rehabilitation of neck pain over the past 25 + years has had little effect on its overall global burden [3, 4]. Neck pain results in high healthcare costs [5], lost work days, reduced productivity [6] and early work exit [7]. Those with chronic neck pain experience poorer quality of life, and have more comorbid conditions and psychological distress than those without pain.[8] One type of neck pain has been termed idiopathic neck pain as the underlying mechanisms remain largely unknown and associated pathological abnormalities have not been consistently identified with current imaging applications [9]. The precise reasons why some patients recover and others continue to report persistent pain are unknown. This highlights a need for renewed innovations, diagnostics, and effective strategies to identify and mitigate risks and associated costs of persistent neck pain.

Muscle size and composition are potential factors that may provide insight into mechanisms underlying idiopathic neck pain or its chronicity, as muscle function has been linked to the onset [10] and persistence of neck pain [11]. Aspects of muscle size include muscle volume; muscle fat infiltrate (MFI) and relative volume represent composition. Muscle volume is quantified by measuring a muscle’s cross-sectional area using imaging, typically MRI, and extrapolating to volume based on MRI slice thickness. MFI and relative volume are non-invasively quantified using pixel intensity from water and fat images derived from multi-echo MRI acquisitions (e.g. Dixon technique) [12–15]. Relative volume represents the size or amount of muscle within the muscle volume that is not identified as MFI. Muscle volume has been shown to be increased in individuals with neck pain from whiplash-associated disorders as compared to healthy controls, with the larger volume made up by MFI [16]. Muscle volume in individuals with idiopathic neck pain has been studied using ultrasound (e.g., [17–19]) with only one study (*n* = 20 with idiopathic neck pain) identified using MRI [20]. Few studies have examined the MFI of cervical muscles, and we identified only one reported dataset of MFI in individuals with chronic idiopathic neck pain [21, 22]. In this cohort of 23 females, MFI was not present in the cervical extensors to the extent that had been reported in individuals with whiplash-associated disorder. The studies that report MFI in females with idiopathic neck pain either reported an overall fat score not identifying specific muscles [21], or limited their measurement to specific spinal levels (C1/2, C2/3 and C5/6) [22]. Hence, investigations of MFI in the cervical musculature of individuals with chronic idiopathic neck pain are limited. Idiopathic neck pain is likely to have different underlying mechanisms to whiplash-associated disorder. Thus, there is a need to investigate MFI more comprehensively in this patient group, specifically across the length of the cervical spine and including a breadth of muscles. Understanding muscle composition in individuals with neck pain may contribute to the development of prognostic and predictive tools to identify patients most likely to progress to chronicity and guide treatment decisions.

The aim of this study was to determine whether there are differences in muscle volume, relative volume and MFI in individuals with chronic idiopathic neck pain compared with age- and sex-matched asymptomatic controls. Six muscles/muscle groups from C3 through T1 were included. Evidence for muscle composition alterations in individuals with idiopathic neck pain may suggest possible muscle degeneration or pathophysiological processes that may identify individuals who require specialised intervention.

## Methods

### Design

This cross-sectional observational study compared the muscle volume, relative volume and the percentage of MFI of cervical extensor and flexor muscles (from C3 to T1) between participants with chronic idiopathic neck pain and asymptomatic age and sex-matched controls. Individuals with chronic idiopathic neck pain (> 3 months) were recruited from a regional city in Australia from the local community via advertisement. Each participant attended two data collection sessions, one where they had clinical measurements conducted, including self-report questionnaires and cervical range of motion, and a second session where they had an MRI examination of the cervical spine. Two blinded researchers (SS, HJT) who did not participate in data collection contoured muscle borders in Analyze Pro (Analyze Direct, Inc., Overland Park, KS, USA) to quantify muscle volume and subsequently relative volume and MFI from MRI. This research was performed according to the Declaration of Helsinki and the study was approved by the Human Research Ethics Committee of the University of Newcastle (H-2015-0235). Informed consent was gained prior to data collection.

### Participants

Eligible participants were able to undergo an MRI exam (no metallic implants, pacemakers, or claustrophobia, not pregnant), and were 18–55 years of age. Those with chronic idiopathic neck pain (> 3 months) had at least 4/10 on a numerical pain rating scale and pain that at least “moderately” interfered with normal work (including housework, from the SF-12) [23]. Asymptomatic participants had no neck or back pain for which they sought treatment in the previous 2 years, no previous history of neck injury/trauma, no current musculoskeletal pain in any body area, and were matched to a pain participant in sex and age (± 5 years). Excluded from both groups were those with headaches as their primary complaint, dizziness, history of neck trauma, neck surgery, diabetes or peripheral vascular disease.

Age, sex, height (cm using a standard stadiometer), weight (kg using a standard scale: Seca, Model 7,621,019,009), body mass index (BMI), physical activity level (Godin Shepherd Leisure-time Physical Activity Questionnaire [24]), and depression (Center for Epidemiologic Studies Short Depression Scale [CES-D 10] [25]) were collected for all participants. Those with pain also recorded their neck disability (Neck Disability Index, NDI [26]), duration of pain (months) and pain intensity (100 mm visual analogue scale [VAS] anchored by ‘no pain’ on the left and ‘worst pain imaginable’ on the right). Pain intensity was quantified for three different recall periods: current, average over the previous 24 h and over the previous four weeks [27]. All participants had their neck range of motion measured using the Cervical Range of Motion instrument (CROM, Performance Attainment Associates, Minnesota, IL, USA) [28], Participants were instructed to move their head as far as possible and each movement direction (flexion, extension, right and left rotation) was repeated 3 times and averaged. These variables (excluding age, sex and BMI) were collected to contextualise the participant sample and were not used in the analysis of between-group differences in the primary outcome measures described below.

### Outcome measures

#### Primary outcomes

Muscle volume, relative volume and MFI were measured from MR images from the intervertebral disc of C2/3 through the intervertebral disc of T1/2. MRI was undertaken on a Siemens Magnetom Prisma 3 Tesla scanner with a 64-channel head/neck array coil. An axial, VIBE (T1-weighted gradient echo) using two-point Dixon technique (Dixon-VIBE) (TR/TE1/TE2 7.05/2.46/3.69ms) was undertaken with a 320 × 320 mm field of view and 448 × 448 acquisition matrix (0.7 mm in-plane resolution) with a slice thickness of 3 mm. A single slab with 52 slices was acquired covering the cephalad portion of C3 through the caudal portion of the T2 vertebral end plate in a scan duration of 6:23 min. Axial slices were aligned parallel to the C2/3 intervertebral disc allowing MRI slices to perpendicularly intersect muscles. The radiographer positioned the head in approximately neutral, using the same coil for every participant to standardize alignment. A foam pad was placed under the head for participant comfort and their head was secured on either side with additional padding to minimize head movement. The radiographer ensured the participant remained stationary by observing them on a monitor.

#### Muscle border identification

Prior to muscle border contouring on MR images, the location of each axial slice in relation to the cervical vertebrae was identified by assigning each slice to a specific spinal level using visualization of its location on a sagittal localizer view. Individual slices were assigned to vertebral levels by first identifying the slice closest to the midsection of each intervertebral disc. Slices between these were assigned to their corresponding vertebral levels using the same sagittal view. Subsequently, the slices identified as traversing through the intervertebral disc were assigned to the spinal level cephalad of the disc. Muscle volume was quantified by manually tracing the fascial boundary of selected neck muscles using a computer mouse on every second MRI slice collected. Automated interpolation of the remaining slices was performed in Analyze Pro (Analyze Pro 1.0, AnalyzeDirect Inc., Overland Park, KS, USA). Interpolation accuracy was checked by visual examination of all slices and three-dimensional models. Errors were re-contoured manually as necessary.

**Fig. 1:**
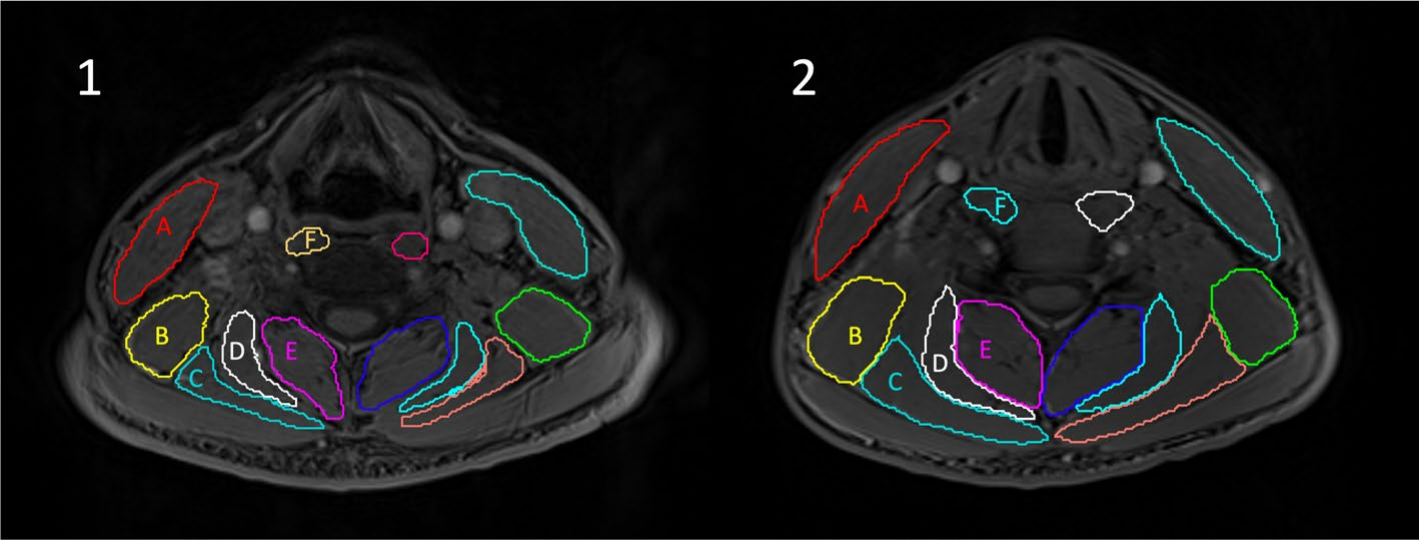
Example of muscle segmentation for a participant with chronic idiopathic neck pain (**1**) and an age and sex-matched asymptomatic control (**2**) at the C5 spinal level. A: sternocleidomastoid, B: levator scapulae, C: splenius capitis (including splenius cervicis), D: semispinalis capitis, E: multifidus (including semispinalis cervicis), and F: longus colli

Muscles were identified, where present, from the most cephalad slice allocated to C3 through to the most caudal slice allocated to T1 on both left and right. Muscles included were the levator scapulae, multifidus including semispinalis cervicis (MFSS), semispinalis capitis, splenius capitis including splenius cervicis (SCSC), sternocleidomastoid and longus colli. These muscles encompass all major deep and superficial lower cervical extensor muscles, as well as two flexors, one deep flexor (longus colli) and one superficial flexor/rotator (sternocleidomastoid). Poorly visualized fascial borders between multifidus and semispinalis cervicis and between splenius capitis and cervicis meant that these muscle pairs were combined to reduce measurement error. Muscles were differentiated with reference to an MRI anatomical atlas outlining the muscles at each level [29]. MFI was identified by calculating the percentage of the MR signal from fat using the fat and water images from the T1 -weighted Dixon images. MFI was calculated for each muscle region of interest on each MRI slice between the top endplate of C3 and the bottom endplate of T1. MFI was defined as the mean fat-only signal within a defined region of interest divided by the sum of the mean fat-only and the mean water-only signals within the same region of interest, multiplied by 100 (MFI = [Fat/(Fat + Water)] x 100) [30]. The fat-only and water-only images were derived from a two-point Dixon technique MRI acquisition (described above within the Methods). Relative volume was calculated by subtracting the MFI percentage from 100, and multiplying the volume by this percent, thus representing the percentage of the segmented volume that can be attributed to muscle. These calculations provided values for each MR slice which were used in the statistical analyses. Figure 1 illustrates the muscle segmentation.

### Statistical analysis

#### Sample size

Our previous work suggested an average within-group SD for MFI across spinal levels of 8.3% [20]. Thus we estimated we could detect a 5% between-group difference (two-tailed independent t-test) in MFI with 44 participants per group, with 80% power and an alpha of 0.05.

#### Data analysis

Inter-rater reliability of muscle segmentation was determined from volume and MFI measures from a sample of 13 participants traced by two researchers (SS, HJT), using intra-class correlation coefficients (methods previously described [31]). The participants were selected to represent a range of ages and equal representation of the sexes. Participant characteristics and unadjusted means for volume and MFI are reported using descriptive statistics. The total number of axial slices varied between participants due to differences in neck length between C3 and T1. Therefore, rather than sum the values from axial slices, which would be affected by neck length, we analysed each value from each axial slice using linear mixed models to account for the repeated measures for each participant. Mixed models are robust to variations in the number of repeated measures between participants.

Bonferroni-adjusted estimated marginal means from linear mixed effects regression models determined differences between groups in muscle volume, relative volume and MFI. Models were adjusted for side (left or right), muscle (levator scapulae, MFSS, semispinalis capitis, SCSC, sternocleidomastoid, longus colli), spinal level, sex, age, and BMI. For categorical variables, the reference category is provided for interpretation of the models [32]. As models were analysed by MRI slice, and each participant had data from multiple slices, we included a random effect for participant. Two-way interactions between muscle and group were included. Statistical analyses were performed in IBM SPSS Version 26.0 (IBM Corporation, Armonk, NY).

## Results

### Participants

Participants with neck pain were recruited from May 25, 2015, through November 26, 2015, with asymptomatic matched controls recruited through May 31, 2017. Of 193 volunteers with neck pain screened, 48 met the inclusion criteria and completed a scanning session. The reasons for exclusion were a history of whiplash or trauma (30%, *n* = 43), migraines (15%, *n* = 22), age > 55 years (12%, *n* = 17), did not meet pain criteria, usually with pain levels too low (12%, *n* = 18), neuropathic pain or fibromyalgia (6%, *n* = 8), reports of dizziness of unknown origin (2%, *n* = 3), not contactable after inquiring about the study (7%, *n* = 10), unable to make an appointment time or declined participation (13%, *n* = 19), other (3%, *n* = 5, e.g., diabetic, congenital fused vertebrae, claustrophobic). One scan was unusable due to motion artefact resulting in 47 participants with pain for analysis. Asymptomatic volunteers (*n* = 35) were enrolled when their age (within 5 years) and sex matched a pain participant. Characteristics of enrolled participants are reported in Table 1. Participants with pain had a mean age of 36.8 (SD 9.8), body mass index (BMI) 25.6 (SD 4.2), mild pain (VAS 30.3, SD 17.7) and neck disability (mean NDI 13.3, SD 4.2) and a long duration of neck pain (mean 67.7 months, SD 59.4). The pain group had less cervical range of motion in all measured directions (Table 1).

**Table 1:** Characteristics of participants

	All	Groups		Difference between groups	
Characteristic	(*n* = 82)	Pain (*n* = 47)	Asymptomatic (*n* = 35)	Pain minus Asymptomatic	P
Age *(yr)*, mean (SD)	36.3 (10.5)	36.8 (9.8)	35.7 (11.5)	1.07 (-3.7 to 5.9)	0.657
Sex *(female)*, number (%)	36 (44)	22 (47)	14 (40)	χ2 = 0.378	0.539
Weight *(kg)*, mean (SD)	75.6 (15.6)	77.0 (15.2)	73.8 (16.2)	3.2 (-3.8 to 10.2)	0.372
Height *(cm)*, mean (SD)	171.9 (10.9)	173.3 (10.8)	169.8 (10.9)	3.5 (-1.3 to 8.4)	0.351
BMI *(kg/m*^*2*^*)*, mean (SD)	25.5 (4.5)	25.6 (4.2)	25.6 (4.8)	0.007 (-2.0 to 2.0)	0.994
Physical activity *(category)*, number (%)					
Insufficiently active	14 (17)^a^	9 (20)	5 (15)	χ2 = 0.623	0.732
Moderately active	16 (20)	8 (18)	8 (24)		
Active	49 (60)	28 (62)	21 (62)		
CES-D 10^b^ *(category)*, number (%)					
Depressed	16 (20)	14 (32)	2 (6)	(Fisher’s exact)	0.005
Not depressed	62 (76)	30 (68)	32 (94)		
Pain intensity, 0-100 mm visual analogue scale *(mm)*, mean (SD)					
Current	–	30.3 (17.7)	–	–	–
24 h recall	–	36.3 (17.9)	–	–	–
4 week recall	–	42.9 (19.0)	–	–	–
Neck Disability Index (*0–50*), mean (SD)	–	13.3 (4.9)	–	–	–
Duration of neck pain *(months)*, mean (SD)	–	67.7 (59.4)	–	–	–
Duration of neck pain *(category)*, number (%)					
3 to 12 months	–	7 (14.9)	–	–	–
1 to 5 years	–	17 (36.2)	–	–	–
5 + years	–	23 (48.9)	–	–	–
Reported radiculopathy, number (%)	–	8 (17.0)	–	–	–
Neck flexion ROM *(°)*, mean (SD)	54.2 (11.2)	48.8 (10.0)	61.6 (8.1)	-12.8 (-17.0 to -8.7)	< 0.001
Neck extension ROM *(°)*, mean (SD)	62.4 (12.5)	60.0 (12.7)	65.8 (11.6)	-5.8 (-11.3 to -0.3)	0.040
Neck right rotation ROM *(°)*, mean (SD)	64.7 (9.9)	61.4 (10.6)	69.3 (6.4)	-7.9 (-11.7 to -4.1)	< 0.001
Neck left rotation ROM *(°)*, mean (SD)	64.4 (9.3)	61.6 (9.7)	68.3 (7.0)	-6.7 (-10.4 to -3.1)	< 0.001

^a^Percentages may not sum to 100 where there is missing data

^b^Center for Epidemiologic Studies Short Depression Scale

### Muscle size and composition

**Fig. 2:**
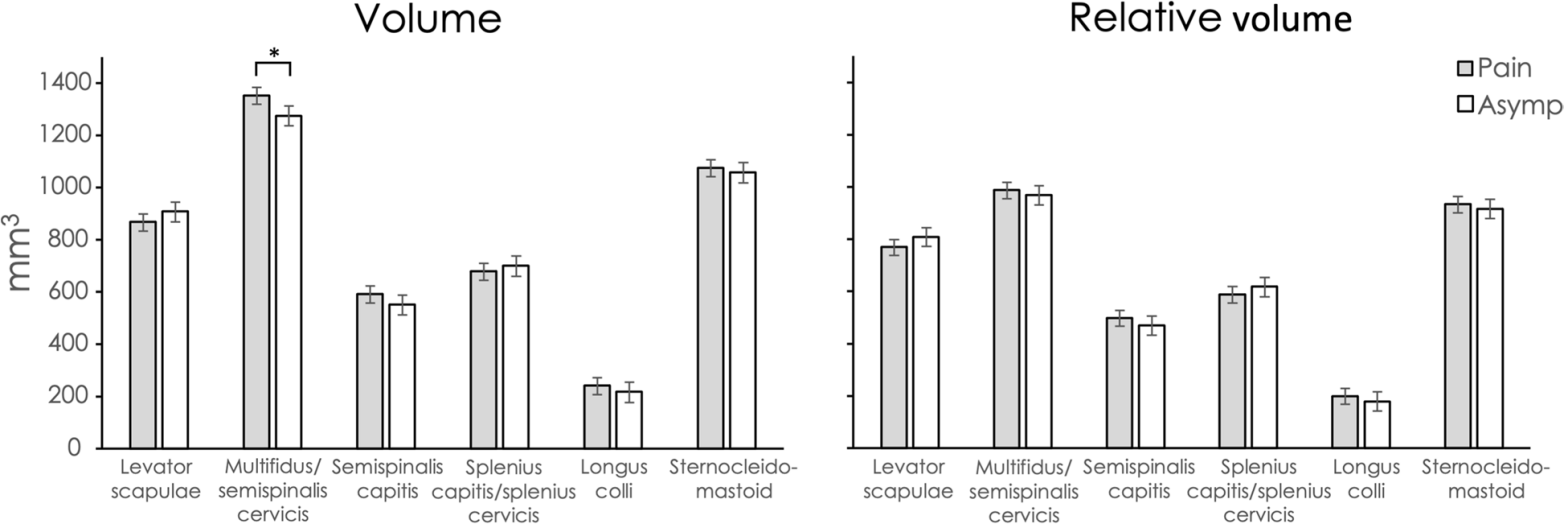
Comparisons of muscle volume and relative volume between individuals with chronic idiopathic neck pain and sex-matched asymptomatic controls from mixed model post-hoc tests adjusted by side (left or right), muscle, spinal level, sex, age, and body mass index (BMI). * denotes statistical significance *p* = .003

**Fig. 3:**
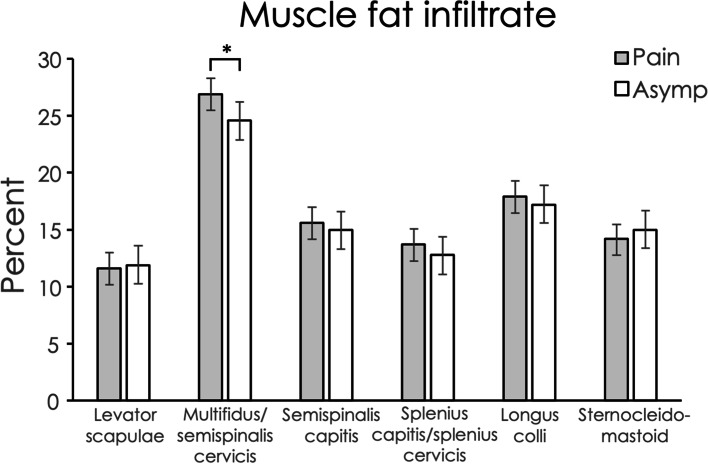
Comparisons of muscle fat infiltrate between individuals with chronic idiopathic neck pain and sex-matched asymptomatic controls from mixed model post-hoc tests adjusted by side (left or right), muscle, spinal level, sex, age, and body mass index (BMI). * denotes statistical significance *p* = .034

Inter-rater reliability of muscle segmentation was excellent for muscle volume (ICC2,1 = 0.97; 95% CI 0.95, 0.98, across all muscles) and MFI (0.85; 0.79, 0.89). Unadjusted mean values for muscle volume, relative volume and MFI for each muscle at each spinal level for each group are reported in Tables S1, S2 and S3 in the Supplementary Information file. Regression modelling showed that being female was associated with a lower muscle volume, higher BMI was associated with greater muscle volume, and muscle volume differed between spinal levels and between muscles (Table 2). The left side had on average less muscle volume than the right. Age was not associated with muscle volume (Table 2). Relative volume was not significantly different between those with and without pain (Table 3). Similar to muscle volume, relative volume was less for females compared to males, less on the left compared to the right, and older age was associated with less relative volume. BMI was not associated with relative volume (Table 3). Table 4 shows that older age and higher BMI were associated with greater MFI, and MFI differed between spinal levels and between muscles. Greater MFI was observed at more caudal spinal levels compared to cephalad levels (Table S3). Sex was not associated with MFI. The left side had on average less MFI than the right, but the difference was small. For muscle volume, relative volume and MFI, there were interactions between muscle type and group suggesting between-group differences varied by muscle. Post-hoc tests showed that when accounting for possible confounders (side, muscle, spinal level, age, sex, BMI), individuals with pain had a greater muscle volume and greater MFI for the MFSS, with no between-group difference in relative volume (Table 5; Figs. 2 and 3).

**Table 2:** Results of linear mixed model investigating the relationship between muscle volume (mm^3^) and group (pain vs. asymptomatic), accounting for side (left or right), muscle, spinal level (C3-T1), sex, age, body mass index (BMI).

Variable	Reference category^a^	Estimate (95% CI)	Std Error	P
Group	Asymptomatic	17.01 (-32.85 to 66.87)	25.10	0.500
Side (left)	Right	-13.62 (-19.96 to -7.28)	3.23	< 0.001
Muscle (levator scapulae)	SCM^c^	-149.86 (-166.76 to -132.97)	8.62	< 0.001
Muscle (multifidus)^b^	SCM	217.24 (199.61 to 234.87)	9.00	< 0.001
Muscle (semispinalis capitis)	SCM	-505.37 (-522.26 to -488.48)	8.62	< 0.001
Muscle (splenius capitis)^b^	SCM	-357.89 (-374.78 to 341.00)	8.62	< 0.001
Muscle (longus colli)	SCM	-840.89 (-857.80 to -823.99)	8.63	< 0.001
Spinal level (C4)	C3	7.77 (-3.40 to 18.93)	5.70	0.173
Spinal level (C5)	C3	23.08 (11.80 to 34.36)	5.76	< 0.001
Spinal level (C6)	C3	40.42 (29.20 to 51.65)	5.73	< 0.001
Spinal level (C7)	C3	-19.97 (-31.04 to -8.89)	5.65	< 0.001
Spinal level (T1)	C3	-257.28 (-268.09 to -246.46)	5.52	< 0.001
Sex (Female)	Male	-293.55 (-343.88 to -243.21)	25.27	< 0.001
Age	–	-2.21 (-4.68 to 0.25)	1.24	0.078
BMI	–	8.75 (3.13 to 14.36)	2.82	0.003
Muscle (levator scapulae)*group(pain)	SCM/asymptomatic	-57.78 (-79.83 to -35.73)	11.25	< 0.001
Muscle (multifidus)*group(pain)	SCM/asymptomatic	59.79 (36.86 to 82.73)	11.70	< 0.001
Muscle (semispinalis capitis)*group(pain)	SCM/asymptomatic	21.77 (-0.28 to 43.82)	11.25	0.053
Muscle (splenius capitis)*group(pain)	SCM/asymptomatic	-39.12 (-61.17 to -17.07)	11.25	0.001
Muscle (longus colli)*group(pain)	SCM/asymptomatic	6.51 (-15.55 to 28.58)	11.27	0.563

^a^The reference category is the category against which the others are compared

^b^Multifidus includes semispinalis cervicis; splenius capitis includes splenius cervicis

^c^SCM, sternocleidomastoid

**Table 3:** Results of linear mixed model investigating the relationship between relative muscle volume (mm^3^) and group (pain vs. asymptomatic), accounting for side (left or right), muscle, spinal level (C3-T1), sex, age, body mass index (BMI).

Variable	Reference category^a^	Estimate (95% CI)	Std Error	P
Group	Asymptomatic	16.74 (-31.50 to 64.98)	24.27	0.492
Side (left)	Right	-8.96 (-14.47 to -3.46)	2.81	0.001
Muscle (levator scapulae)	SCM^c^	-106.57 (-121.24 to -91.90)	7.48	< 0.001
Muscle (multifidus)^b^	SCM	53.10 (37.79 to 68.41)	7.81	< 0.001
Muscle (semispinalis capitis)	SCM	-445.83 (-460.49 to -431.16)	7.48	< 0.001
Muscle (splenius capitis)^b^	SCM	-297.69 (-312.37 to -283.02)	7.49	< 0.001
Muscle (longus colli)	SCM	-737.18 (-751.86 to -722.49)	7.49	< 0.001
Spinal level (C4)	C3	24.91 (15.22 to 34.61)	4.95	< 0.001
Spinal level (C5)	C3	51.16 (41.37 to 60.96)	5.00	< 0.001
Spinal level (C6)	C3	61.42 (51.68 to 71.17)	4.97	< 0.001
Spinal level (C7)	C3	-9.02 (-18.64 to 0.60)	4.91	0.066
Spinal level (T1)	C3	-223.62 (-233.02 to -214.23)	4.79	< 0.001
Sex (Female)	Male	-262.85 (-311.96 to -213.75)	24.65	< 0.001
Age	–	-2.70 (-5.10 to -0.29)	1.21	0.028
BMI	–	3.88 (-1.60 to 9.36)	2.75	0.162
Muscle (levator scapulae)*group(pain)	SCM/asymptomatic	-57.00 (-76.14 to -37.86)	9.77	< 0.001
Muscle (multifidus)*group(pain)	SCM/asymptomatic	0.66 (-19.25 to 20.58)	10.16	0.948
Muscle (semispinalis capitis)*group(pain)	SCM/asymptomatic	10.91 (-8.24 to 30.05)	9.77	0.264
Muscle (splenius capitis)*group(pain)	SCM/asymptomatic	-46.92 (-66.07 to -27.77)	9.77	< 0.001
Muscle (longus colli)*group(pain)	SCM/asymptomatic	3.30 (-15.86 to 22.47)	9.78	0.735

^a^The reference category is the category against which the others are compared

^b^Multifidus includes semispinalis cervicis; splenius capitis includes splenius cervicis

^c^SCM, sternocleidomastoid

**Table 4:** Results of linear mixed model investigating the relationship between muscle fat infiltrate (as a percent of muscle volume) and group (pain vs. asymptomatic), accounting for side (left or right), muscle, spinal level (C3-T1), sex, age, body mass index (BMI).

Variable	Reference category^a^	Estimate (95% CI)	Std Error	P
Group	Asymptomatic	-0.87 (-3.01 to 1.26)	1.07	0.418
Side (left)	Right	-0.36 (-0.46 to -0.25)	0.05	< 0.001
Muscle (levator scapulae)	SCM^c^	-3.09 (-3.37 to -2.82)	0.14	< 0.001
Muscle (multifidus)^b^	SCM	9.53 (9.24 to 9.81)	0.15	< 0.001
Muscle (semispinalis capitis)	SCM	-0.06 (-0.36 to 0.21)	0.14	0.667
Muscle (splenius capitis)^b^	SCM	-2.27 (-2.54 to -1.99)	0.14	< 0.001
Muscle (longus colli)	SCM	2.21 (1.94 to 2.49)	0.14	< 0.001
Spinal level (C4)	C3	-1.28 (-1.46 to -1.10)	0.09	< 0.001
Spinal level (C5)	C3	-2.15 (-2.34 to -1.97)	0.09	< 0.001
Spinal level (C6)	C3	-1.71 (-1.90 to -1.53)	0.09	< 0.001
Spinal level (C7)	C3	-0.03 (-0.21 to 0.15)	0.09	0.728
Spinal level (T1)	C3	3.10 (2.92 to 3.27)	0.09	< 0.001
Sex (Female)	Male	2.00 (-0.23 to 4.23)	1.12	0.078
Age	–	0.11 (0.004 to 0.22)	0.05	0.043
BMI	–	0.44 (0.19 to 0.69)	0.12	0.001
Muscle (levator scapulae)*group(pain)	SCM/asymptomatic	0.56 (0.20 to 0.92)	0.18	0.002
Muscle (multifidus)*group(pain)	SCM/asymptomatic	3.19 (2.82 to 3.57)	0.19	< 0.001
Muscle (semispinalis capitis)*group(pain)	SCM/asymptomatic	1.52 (1.16 to 1.88)	0.18	< 0.001
Muscle (splenius capitis)*group(pain)	SCM/asymptomatic	1.80 (1.44 to 2.15)	0.18	< 0.001
Muscle (longus colli)*group(pain)	SCM/asymptomatic	1.52 (1.17 to 1.88)	0.18	< 0.001

^a^The reference category is the category against which the others are compared

^b^Multifidus includes semispinalis cervicis; splenius capitis includes splenius cervicis

^c^SCM, sternocleidomastoid

**Table 5:** Estimated marginal means (95% CI) for muscle volume (mm^3^), relative volume (mm^3^) and muscle fat infiltrate (expressed as a percentage of muscle volume) for each muscle from linear mixed models adjusted by side (left /right), muscle, spinal level, gender, age, and BMI, with Bonferroni-adjusted mean differences between groups

Characteristic	Groups		Difference between groups	
	Pain(*n* = 47)	Asymp*(*n* = 35)	Pain minus Asymp	P
Volume				
Levator scapulae	867.9 (835.5 to 900.2)	908.6 (870.5 to 946.8)	-40.8 (-90.6 to 9.1)	0.108
Multifidus (with semispinalis cervicis)	1352.5 (1320.0 to 1385.1)	1275.7 (1237.3 to 1314.2)	76.8 (26.6 to 127.0)	0.003
Semispinalis capitis	591.9 (559.6 to 624.2)	553.1 (515.0 to 591.2)	38.8 (-11.1 to 88.6)	0.126
Splenius capitis with splenius cervicis	678.5 (646.2 to 710.8)	700.6 (662.5 to 738.7)	-22.1 (-72.0 to 27.7)	0.381
Longus colli	241.1 (208.8 to 273.4)	217.6 (179.5 to 255.7)	23.5 (-26.3 to 73.4)	0.351
Sternocleidomastoid	1075.5 (1043.2 to 1107.8)	1058.5 (1020.3 to 1096.6)	17.0 (-32.8 to 66.9)	0.500
Relative volume				
Levator scapulae	769.18 (737.9 to 800.5)	809.44 (772.6 to 846.3)	-40.3 (-88.5 to 8.0)	0.101
Multifidus (with semispinalis cervicis)	986.51 (955.0 to 1018.0)	969.11 (932.0 to 1006.2)	17.4 (-31.1 to 65.9)	0.478
Semispinalis capitis	497.8 (466.5 to 529.1)	470.18 (433.3 to 507.1)	27.6 (-20.6 to 75.9)	0.258
Splenius capitis with splenius cervicis	588.14 (556.9 to 619.4)	618.3 (581.4 to 655.2)	-30.2 (-78.4 to 18.1)	0.217
Longus colli	198.9 (167.6 to 230.2)	178.8 (141.9 to 215.7)	20.0 (-28.2 to 68.3)	0.411
Sternocleidomastoid	932.7 (901.5 to 964.0)	916.0 (879.1 to 952.9)	16.7 (-31.5 to 65.0)	0.492
Muscle fat infiltrate				
Levator scapulae	11.6 (10.2 to 13.0)	11.9 (10.3 to 13.6)	-0.3 (-2.4 to 1.8)	0.769
Multifidus (with semispinalis cervicis)	26.9 (25.5 to 28.3)	24.6 (22.9 to 26.2)	2.3 (0.2 to 4.5)	0.034
Semispinalis capitis	15.6 (14.2 to 17.0)	15.0 (13.3 to 16.6)	0.6 (-1.5 to 2.8)	0.547
Splenius capitis with splenius cervicis	13.7 (12.3 to 15.1)	12.8 (11.1 to 14.4)	0.9 (-1.2 to 3.1)	0.392
Longus colli	17.9 (16.5 to 19.3)	17.2 (15.6 to 18.9)	0.7 (-1.5 to 2.8)	0.545
Sternocleidomastoid	14.2 (12.8 to 15.5)	15.0 (13.4 to 16.7)	-0.9 (-3.0 to 1.3)	0.418

*Asymptomatic

## Discussion

This study found that individuals with chronic idiopathic neck pain had greater muscle volume and MFI in their deep extensor muscles (MFSS) as compared to age and sex-matched controls. This difference was apparent, even when accounting for differences in age and BMI, factors believed to affect MFI [22, 33, 34]. MFI values differed depending on the spinal level measured, with more caudad spinal levels generally displaying greater MFI than more cephalad levels between C3 and TI. MFI differed between muscles, with the MFSS having the highest MFI. The MFSS had a larger muscle volume with greater MFI in individuals with chronic idiopathic neck pain compared to controls, with no between-group difference in relative volume. As relative volume represents the muscle volume excluding the fat infiltrate, this suggests that lean muscle mass is similar between the pain and asymptomatic groups. Notably, the between-group difference in MFI was small and its clinical relevance is unknown. Nevertheless, MFI may be one factor that may identify individuals with chronic idiopathic neck pain. The development of clinical tools to identify MFI in individuals with chronic pain is needed to establish the clinical relevance of MFI. Consistent findings of MFI in individuals with chronic pain may lead to personalised interventions and the ability to direct treatment resources effectively.

Evidence for the clinical correlates of MFI has largely been derived from studies of individuals with low back pain [33–36] or types of neck pain other than idiopathic neck pain (e.g., whiplash associated disorder, cervical myelopathy) [16, 37–41]. These studies have shown that MFI appears to be associated with higher levels of disability in patients with WAD [40, 42] or cervical myelopathy [38]. Greater MFI is associated with postural instability and poor balance in patients with radicular spondylopathy [37]. Functional recovery after surgical decompression is worse with higher MFI [39]. Relationships between clinical findings and MFI are reported more frequently for the multifidus compared to other muscles in both the cervical [38] and lumbar spines [36]. These findings suggest that MFI in the multifidus may be radiologic sign, potentially identifying patients with a less favourable prognosis.

One previous study of MFI volume that included individuals with idiopathic neck pain found that MFI at C2/3 and C5/6 was similar to healthy controls and less than that observed in those with whiplash-associated disorder [22]. That study was limited to females and had a smaller sample of participants with idiopathic neck pain than the current study. It also had a smaller number of participants with idiopathic neck pain than their comparison groups, possibly affecting ability to detect a significant difference. The participants in the current study had a longer duration of neck pain, on average, than the previous study (68 vs. 34 months), possibly accounting for differences in findings. Another study using MRI found differences in muscle cross-sectional area in females with chronic idiopathic neck pain compared to healthy controls, but only their whiplash group showed increases in MFI [43]. This study was limited to selected spinal levels for each measured muscle between C1 and C5, which may have reduced the strength of their analyses. There are also limitations in using cross-sectional area rather than volume for quantifying muscle composition. The lack of studies investigating muscle composition in individuals with idiopathic neck pain suggests more research is warranted.

The current study found that MFI in the MFSS of individuals with idiopathic neck pain was greater than in asymptomatic matched controls, accounting for both age and BMI. Older age is associated with increased MFI in the lumbar spine [33, 34], and this was consistent in the current study. Higher BMI has also been associated with greater MFI in the lumbar [34] and cervical spines [22] previously, and in the current study. After adjusting for age and BMI, the greater MFI in the MFSS remained in those with chronic neck pain. This was consistent when observing the unadjusted mean value for MFI for the MFSS and the values at each spinal level (except C3, Table S3 in the Supplementary Information file). The unadjusted values for the SCSC, semispinalis capitis, and longus colli also showed greater MFI in individuals with neck pain compared to controls overall; at all spinal levels for SCSC, 3 of 6 spinal levels for semispinalis capitis, and 4 of 6 for longus colli (Table S3 in the Supplementary Information file). This may suggest that age and BMI could account for the between-group differences in MFI in those muscles. Alternatively, it may mean the differences between people with and without neck pain were not large enough, or the lack of homogeneity proved a challenge to detect significance. Nonetheless, the greater MFI in the MFSS regardless of age and BMI highlights the complex uniqueness of the multifidus muscle. Indeed, there is evidence that the deep cervical muscles function differently to the superficial muscles during a motor skill task [44].

The multifidus was combined with the semispinalis cervicis for segmentation. Thus there may have been intermuscular fat between the two muscles, potentially accounting for the higher MFI observed in this muscle group. When segmenting the multifidus and semispinalis cervicis, their close approximation and similar attachments of muscle fascicles makes these muscles challenging if not impossible to differentiate in cross-section. To improve accuracy of segmentation and reliability between the two researchers performing the segmentations, we chose to combine multifidus and semispinalis cervicis, as has been recommended in previous research [13, 45] The small between-group difference in MFI of the MFSS may not be clinically relevant. However, it is unlikely to be a chance finding, considering the consistency of findings across spinal levels (Table S3 in the Supplementary Information file) and across muscle volume, relative volume and MFI. Muscle volume was greater in the MFSS of the pain group, with relative volume no different, suggesting the extra muscle volume may consist of MFI.

As the current study was cross-sectional, it cannot determine if MFI is a cause or an effect of pain. There is some evidence that MFI increases in healthy individuals after 4 weeks of immobilisation [46], and 12 weeks of strength training can decrease MFI in the thigh muscles of older individuals [47] These findings suggest that future research should investigate interventions that might have the potential to reduce MFI in the cervical multifidus and semispinalis cervicis to determine any effect on neck pain. In the neck muscles, there is evidence the deep cervical muscles function differently to the superficial muscles: specific exercises preferentially activate the deep semispinalis cervicis over the more superficial splenius capitis [48, 49]. This suggests that specificity will be required to achieve improvements in muscle composition. Importantly, the current findings showed that muscle volume and MFI differed between muscles, and for each muscle, values varied depending on spinal level measured. Thus, studies of muscle size and composition should include as many muscles and spinal levels as is feasible, and studies of single muscles or spinal levels should not be generalized to the health of the entire cervical spine. Future research may determine whether a single spinal level may be able to effectively represent the muscle volume and composition of an individual muscle.

The strengths of this study include the measurement of muscle volumes and MFI from multiple cervical muscles across multiple spinal levels, allowing quantification of the majority of existing muscle covering the cervical spine. This allowed comparisons across muscles and spinal levels. It is, to our knowledge, only the third study to examine these variables in individuals with idiopathic neck pain, and the first to include all spinal levels from C3-T1. Reliability of contouring between researchers was comparable or better than that previously reported using the same methods [31]. Results are limited to this sample of individuals with chronic idiopathic neck pain. Participants in this study reported an average duration of neck pain of 63 months, with half of the sample reporting they had experienced neck pain for greater than five years. It is unknown if changes in MFI might be recognised earlier in individuals who go on to develop persistent neck pain symptoms, potentially enabling targeted interventions.

Future research should develop methods to enable muscle volume and MFI to be quantified in the clinical setting, potentially through automated methods that eliminate the time needed to manually contour muscle boundaries [30]. As MFI varies based on age and BMI, a large normative database is needed to effectively identify deviations from normal. Finally, investigations of interventions that may reduce MFI, such as resistance training or specific muscle retraining, need to be conducted to determine whether MFI and neck pain can both be reduced with intervention.

## Conclusion

In this study, individuals with chronic idiopathic neck pain had greater MFI in the multifidus muscle (combined with the semispinalis cervicis) compared to age and sex-matched asymptomatic controls, while controlling for age and BMI. The between-group difference in MFI was small. Nonetheless, these findings may suggest an underlying neurobiological rationale for chronic idiopathic neck pain that may be a contributor to, or consequence of, neck pain.

## Supplementary Information

**Additional file 1:** **Table S1.** Mean (SD) for unadjusted muscle volume for each group (chronic idiopathic neck pain and asymptomatic) calculated per MRI slice for each muscle at each spinal level, with mean difference (95% CI) between groups. **Table S2.** Mean (SD) for unadjusted relative muscle volume for each group (chronic idiopathic neck pain and asymptomatic) calculated per MRI slice for each muscle at each spinal level, with mean difference (95% CI) between groups. **Table S3.** Mean (SD) for unadjusted MFI (%) for each group (chronic idiopathic neck pain and asymptomatic) calculated per MRI slice for each muscle at each spinal level, with mean difference (95% CI) between groups.

## Data Availability

The datasets generated and/or analysed during the current study are not publicly available due to them containing information that could compromise research participant privacy/consent, but are available from the corresponding author on reasonable request.

## References

[1] Hoy D, March L, Woolf A, Blyth F, Brooks P, Smith E, Vos T, Barendregt J, Blore J, Murray C (2014). The global burden of neck pain: estimates from the Global Burden of Disease 2010 study. Ann Rheum Dis.

[2] Blyth FM, Briggs AM, Schneider CH, Hoy DG, March LM (2019). The global burden of musculoskeletal pain-Where to from here?. Am J Public Health.

[3] Safiri S, Kolahi A-A, Hoy D, Buchbinder R, Mansournia MA, Bettampadi D, Ashrafi-Asgarabad A, Almasi-Hashiani A, Smith E, Sepidarkish M (2020). Global, regional, and national burden of neck pain in the general population, 1990–2017: systematic analysis of the Global Burden of Disease Study 2017. BMJ.

[4] Vos T, Lim SS, Abbafati C, Abbas KM, Abbasi M, Abbasifard M, Abbasi-Kangevari M, Abbastabar H, Abd-Allah F, Abdelalim A (2020). Global burden of 369 diseases and injuries in 204 countries and territories, 1990–2019: a systematic analysis for the Global Burden of Disease Study 2019. The Lancet.

[5] Dieleman JL, Baral R, Birger M, Bui AL, Bulchis A, Chapin A, Hamavid H, Horst C, Johnson EK, Joseph J (2016). US spending on personal health care and public health, 1996–2013. JAMA.

[6] Pereira MJ, Johnston V, Straker LM, Sjogaard G, Melloh M, O’Leary SP, Comans TA (2017). An investigation of self-reported health-related productivity loss in office workers and associations with individual and work-related factors using an employer’s perspective. J Occup Environ Med.

[7] March L, Smith EU, Hoy DG, Cross MJ, Sanchez-Riera L, Blyth F, Buchbinder R, Vos T, Woolf AD (2014). Burden of disability due to musculoskeletal (MSK) disorders. Best Pract Res Clin Rheumatol.

[8] Strine TW, Hootman JM (2007). US national prevalence and correlates of low back and neck pain among adults. Arthritis Rheum.

[9] Rudy IS, Poulos A, Owen L, Batters A, Kieliszek K, Willox J, Jenkins H (2015). The correlation of radiographic findings and patient symptomatology in cervical degenerative joint disease: a cross-sectional study. Chiropr Man Therap.

[10] Shahidi B, Curran-Everett D, Maluf KS (2015). Psychosocial, physical, and neurophysiological risk factors for chronic neck pain: A prospective inception cohort study. J Pain.

[11] Alalawi A, Devecchi V, Gallina A, Luque-Suarez A, Falla D (2022). Assessment of neuromuscular and psychological function in people with recurrent neck pain during a period of remission: Cross-sectional and longitudinal analyses. J Clin Med.

[12] Reeder SB, Hu HH, Sirlin CB (2012). Proton density fat-fraction: a standardized MR-based biomarker of tissue fat concentration. J Magn Reson Imaging.

[13] Elliott JM, Cornwall J, Kennedy E, Abbott R, Crawford RJ (2018). Towards defining muscular regions of interest from axial magnetic resonance imaging with anatomical cross-reference: part II - cervical spine musculature. BMC Musculoskelet Disord.

[14] Elliott JM, Rademaker A, Parrish TB, Walton D (2013). Quantification of cervical spine muscle fat: A comparison between T1-weighted and multi-echo gradient echo imaging using a variable projection algorithm (VARPRO). BMC Med Imaging.

[15] Dixon W (1984). Simple proton spectroscopic imaging. Radiology.

[16] De Pauw R, Coppieters I, Kregel J, De Meulemeester K, Danneels L, Cagnie B (2016). Does muscle morphology change in chronic neck pain patients? - A systematic review. Man Ther.

[17] Javanshir K, Rezasoltani A, Mohseni-Bandpei MA, Amiri M, Ortega-Santiago R, Fernandez-de-Las-Penas C (2011). Ultrasound assessment of bilateral longus colli muscles in subjects with chronic bilateral neck pain. Am J Phys Med Rehabil.

[18] Rahnama L, Rezasoltani A, Zavieh MK, NooriKochi F, Baghban AA (2015). Differences in cervical multifidus muscle thickness during isometric contraction of shoulder muscles: a comparison between patients with chronic neck pain and healthy controls. J Manipulative Physiol Ther.

[19] Rezasoltani A, Ahmadipoor A, Khademi-Kalantari K, Javanshir K (2012). The sign of unilateral neck semispinalis capitis muscle atrophy in patients with chronic non-specific neck pain. J Back Musculoskelet Rehabil.

[20] Snodgrass SJ, Croker C, Yerrapothu M, Shepherd S, Stanwell P, Holder C, Oldmeadow C, Elliott J (2019). Cervical muscle volume in individuals with idiopathic neck pain compared to asymptomatic controls: A cross-sectional magnetic resonance imaging study. Musculoskelet Sci Pract.

[21] Elliott J, Sterling M, Noteboom JT, Darnell R, Galloway G, Jull G (2008). Fatty infiltrate in the cervical extensor muscles is not a feature of chronic, insidious-onset neck pain. Clin Radiol.

[22] Elliott JM, Pedler AR, Jull GA, Van Wyk L, Galloway GG, O’Leary SP (2014). Differential changes in muscle composition exist in traumatic and nontraumatic neck pain. Spine.

[23] Ware J, Kosinski M, Keller SD (1996). A 12-Item Short-Form Health Survey: construction of scales and preliminary tests of reliability and validity. Med Care.

[24] Godin G (2011). The Godin-Shephard Leisure-Time Physical Activity Questionnaire. Health & Fitness Journal of Canada.

[25] Andresen EM, Malmgren JA, Carter WB, Patrick DL (1994). Screening for depression in well older adults: evaluation of a short form of the CES-D (Center for Epidemiologic Studies Depression Scale). Am J Prev Med.

[26] Vernon H, Mior S (1991). The Neck Disability Index: a study of reliability and validity. J Manipulative Physiol Ther.

[27] Kamper SJ, Grootjans SJ, Michaleff ZA, Maher CG, McAuley JH, Sterling M (2014). Measuring pain intensity in patients with neck pain: Does it matter how you do it?. Pain Pract.

[28] Audette I, Dumas J-P, Côté JN, De Serres SJ (2010). Validity and between-day reliability of the Cervical Range of Motion (CROM) Device. J Orthop Sports Phys Ther.

[29] Au J, Perriman DM, Pickering MR, Buirski G, Smith PN, Webb AL. Magnetic resonance imaging atlas of the cervical spine musculature. Clin Anat. 2016:29(5):43–59. 10.1002/ca.22731 27106787

[30] Weber KA, Abbott R, Bojilov V, Smith AC, Wasielewski M, Hastie TJ, Parrish TB, Mackey S, Elliott JM (2021). Multi-muscle deep learning segmentation to automate the quantification of muscle fat infiltration in cervical spine conditions. Sci Rep.

[31] Snodgrass SJ, de Zoete RMJ, Croker C, Yerrapothu M, Elliott JM (2019). Reliability of cervical muscle volume quantification using magnetic resonance imaging. Musculoskelet Sci Pract.

[32] Hosmer DW, Lemeshow S (2000). Applied Logistic Regression.

[33] Crawford RJ, Filli L, Elliott JM, Nanz D, Fischer MA, Marcon M, Ulbrich EJ (2016). Age- and level-dependence of fatty Infiltration in lumbar paravertebral muscles of healthy volunteers. AJNR Am J Neuroradiol.

[34] Peng X, Li X, Xu Z, Wang L, Cai W, Yang S, Liao W, Cheng X (2020). Age-related fatty infiltration of lumbar paraspinal muscles: a normative reference database study in 516 Chinese females. Quant Imaging Med Surg.

[35] Menezes-Reis R, Bonugli GP, Salmon CEG, Mazoroski D, Herrero C, Nogueira-Barbosa MH (2018). Relationship of spinal alignment with muscular volume and fat infiltration of lumbar trunk muscles. PLoS ONE.

[36] Hildebrandt M, Fankhauser G, Meichtry A, Luomajoki H (2017). Correlation between lumbar dysfunction and fat infiltration in lumbar multifidus muscles in patients with low back pain. BMC Musculoskelet Disord.

[37] Mitsutake T, Sakamoto M, Chyuda Y, Oka S, Hirata H, Matsuo T, Oishi T, Horikawa E (2016). Greater cervical muscle fat infiltration evaluated by magnetic resonance imaging is associated with poor postural stability in patients with cervical spondylotic radiculopathy. Spine.

[38] Cloney M, Smith AC, Coffey T, Paliwal M, Dhaher Y, Parrish T, Elliott J, Smith ZA (2018). Fatty infiltration of the cervical multifidus musculature and their clinical correlates in spondylotic myelopathy. J Clin Neurosci.

[39] Paliwal M, Weber KA 2nd, Smith AC, Elliott JM, Muhammad F, Dahdaleh NS, Bodurka J, Dhaher Y, Parrish TB, Mackey S, et al. Fatty infiltration in cervical flexors and extensors in patients with degenerative cervical myelopathy using a multi-muscle segmentation model. PLoS ONE. 2021;16(6):e0253863. 10.1371/journal.pone.0253863 PMC8232539 34170961

[40] Kim CY, Lee SM, Lim SA, Choi YS (2018). Impact of fat infiltration in cervical extensor muscles on cervical lordosis and neck pain: A cross-sectional study. Clin Orthop Surg.

[41] Smith AC, Albin SR, Abbott R, Crawford RJ, Hoggarth MA, Wasielewski M, Elliott JM (2020). Confirming the geography of fatty infiltration in the deep cervical extensor muscles in whiplash recovery. Sci Rep.

[42] Karlsson A, Leinhard OD, Aslund U, West J, Romu T, Smedby O, Zsigmond P, Peolsson A (2016). An investigation of fat infiltration of the multifidus muscle in patients with severe neck symptoms associated with chronic whiplash-associated disorder. J Orthop Sports Phys Ther.

[43] Van Looveren E, Cagnie B, Coppieters I, Meeus M, De Pauw R (2021). Changes in muscle morphology in female chronic neck pain patients using magnetic resonance imaging. Spine.

[44] Röijezon U, Jull G, Djupsjöbacka M, Salomoni SE, Hodges PW (2021). Deep and superficial cervical muscles respond differently to unstable motor skill tasks. Hum Mov Sci.

[45] Abbott R, Pedler A, Sterling M, Hides J, Murphey T, Hoggarth M, Elliott J (2015). The geography of fatty infiltrates within the cervical multifidus and semispinalis cervicis in individuals with chronic whiplash-associated disorders. J Orthop Sports Phys Ther.

[46] Manini TM, Clark BC, Nalls MA, Goodpaster BH, Ploutz-Snyder LL, Harris TB (2007). Reduced physical activity increases intermuscular adipose tissue in healthy young adults. Am J Clin Nutr.

[47] Marcus RL, Addison O, Kidde JP, Dibble LE, Lastayo PC (2010). Skeletal muscle fat infiltration: impact of age, inactivity, and exercise. J Nutr Health Aging.

[48] Rivard J, Unsleber C, Schomacher J, Erlenwein J, Petzke F, Falla D (2017). Activation of the semispinalis cervicis and splenius capitis with cervical pulley exercises. Musculoskelet Sci Pract.

[49] Schomacher J, Erlenwein J, Dieterich A, Petzke F, Falla D (2015). Can neck exercises enhance the activation of the semispinalis cervicis relative to the splenius capitis at specific spinal levels?. Man Ther.

