# Supplementary material for: Greater muscle volume and muscle fat infiltrate in the deep cervical spine extensor muscles (multifidus with semispinalis cervicis) in individuals with chronic idiopathic neck pain compared to age and sex-matched asymptomatic controls: a cross-sectional study
Source: BMC Musculoskelet Disord. 2022 Nov 10;23:973. doi: 10.1186/s12891-022-05924-3 (PMC9647973; doi:10.1186/s12891-022-05924-3)
Supplement: Supplementary file 1 — Additional file 1: Table S1. Mean (SD) for unadjusted muscle volume for each group (chronic idiopathic neck pain and asymptomatic) calculated per MRI slice for each muscle at each spinal level, with mean difference (95% CI) between groups. Table S2. Mean (SD) for unadjusted relative muscle volume for each group (chronic idiopathic neck pain and asymptomatic) calculated per MRI slice for each muscle at each spinal level, with mean difference (95% CI) between groups. Table S3. Mean (SD) for unadjusted MFI (%) for each group (chronic idiopathic neck pain and asymptomatic) calculated per MRI slice for each muscle at each spinal level, with mean difference (95% CI) between groups. [file 12891_2022_5924_MOESM1_ESM.docx]

**SUPPLEMENTARY TABLES**

**TABLE S1.** Mean (SD) for unadjusted muscle volume for each group (chronic idiopathic neck pain and asymptomatic) calculated per MRI slice for each muscle at each spinal level, with mean difference (95% CI) between groups.

| Muscle volume *(mm^3^)* | All | Groups | | | Difference between groups |  |
| --- | --- | --- | --- | --- | --- | --- |
|  | (n=82) | Pain (n=47) | Asymp (n=35) |  | Pain minus Asymp | P |
|  |  |  |  |  |  |  |
| Levator scapulae |  |  |  |  |  |  |
| C3 | 464.3 (212.6) | 433.1 (193.3) | 506.2 (229.7) |  | -73.1 (-99.3 to -47.0) | <.001 |
| C4 | 714.1 (295.5) | 681.9 (263.8) | 756.7 (328.3) |  | -74.8 (-113.8 to -35.7) | <.001 |
| C5 | 975.5 (365.4) | 932.9 (329.1) | 1036.3 (404.5) |  | -103.4 (-153.4 to -53.5) | <.001 |
| C6 | 1220.0 (495.1) | 1180.9 (455.2) | 1273.8 (541.3) |  | -92.8 (-159.4 to -26.3) | .006 |
| C7 | 1302.9 (539.6) | 1265.4 (519.6) | 1355.5 (562.9) |  | -90.1 (-160.1 to -20.2) | .012 |
| T1 | 876.6 (419.3) | 838.2 (410.3) | 929.3 (426.2) |  | -91.1 (-141.7 to -40.6) | <.001 |
| Total | 914.7 (494.6) | 879.9 (473.6) | 962.4 (518.4) |  | -82.5 (-108.4 to -56.5) | <.001 |
| Multifidus (with semispinalis cervicis) |  |  |  |  |  |  |
| C3 | 1137.2 (262.4) | 1165.7 (278.1) | 1093.5 (230.9) |  | 72.3 (10.0 to 134.5) | .023 |
| C4 | 1235.6 (253.3) | 1258.4 (265.6) | 1205.8 (233.1) |  | 52.7 (20.2 to 85.2) | .002 |
| C5 | 1369.5 (314.0) | 1372.2 (316.5) | 1365.6 (310.7) |  | 6.6 (-35.2 to 48.4) | .757 |
| C6 | 1507.5 (369.4) | 1537.1 (372.6) | 1466.5 (361.4) |  | 70.6 (22.3 to 118.9) | .004 |
| C7 | 1440.2 (366.9) | 1461.0 (372.9) | 1411.1 (356.8) |  | 49.9 (2.9 to 96.8) | .037 |
| T1 | 1212.2 (342.7) | 1237.1 (348.5) | 1178.0 (332.0) |  | 59.1 (17.6 to 100.5) | .005 |
| Total | 1337.4 (351.1) | 1358.4 (356.8) | 1308.4 (341.1) |  | 49.9 (30.3 to 69.5) | <.001 |
| Semispinalis capitis |  |  |  |  |  |  |
| C3 | 1081.3 (390.5) | 1097.0 (399.6) | 1060.2 (377.4) |  | 36.8 (-10.7 to 84.2) | .129 |
| C4 | 864.4 (323.9) | 881.0 (333.1) | 842.5 (310.5) |  | 38.5 (-2.9 to 79.9) | .068 |
| C5 | 633.8 (261.2) | 644.8 (269.5) | 618.1 (248.4) |  | 26.6 (-8.1 to 61.4) | .132 |
| C6 | 428.3 (182.0) | 433.6 (182.4) | 420.9 (181.6) |  | 12.7 (-11.2 to 36.6) | .297 |
| C7 | 327.0 (148.8) | 328.6 (147.6) | 324.7 (150.6) |  | 3.9 (-15.1 to 23.0) | .685 |
| T1 | 242.9 (116.0) | 241.5 (118.1) | 244.8 (113.1) |  | -3.2 (-17.3 to 10.8) | .652 |
| Total | 597.9 (400.0) | 604.2 (407.8) | 589.4 (389.0) |  | 14.8 (-5.9 to 35.6) | .162 |
| Splenius capitis (with splenius cervicis) |  |  |  |  |  |  |
| C3 | 976.9 (341.4) | 966.4 (321.9) | 990.9 (365.9) |  | -24.5 (-66.8 to 17.8) | .256 |
| C4 | 856.0 (278.0) | 827.5 (252.1) | 893.7 (305.2) |  | -66.2 (-102.8 to -29.6) | <.001 |
| C5 | 743.5 (234.1) | 724.5 (215.5) | 770.7 (256.2) |  | -46.3 (-78.2 to -14.3) | .005 |
| C6 | 671.7 (209.5) | 650.9 (186.8) | 700.4 (234.6) |  | -49.5 (-77.8 to -21.1) | .001 |
| C7 | 568.4 (208.2) | 548.5 (186.9) | 596.1 (232.1) |  | -47.5 (-75.0 to -20.0) | .001 |
| T1 | 447.4 (176.2) | 435.5 (167.0) | 463.8 (187.1) |  | -28.3 (-50.0 to -6.6) | .011 |
| Total | 709.7 (307.1) | 690.6 (290.9) | 735.8 (326.4) |  | -45.3 (-61.4 to -29.1) | <.001 |
| Sternocleidomastoid |  |  |  |  |  |  |
| C3 | 1009.4 (330.7) | 1004.8 (345.1) | 1015.6 (310.7) |  | -10.8 (-50.4 to 28.8) | .593 |
| C4 | 1265.1 (367.6) | 1242.4 (373.2) | 1295.1 (358.4) |  | -52.8 (-100.1 to -5.4 | .029 |
| C5 | 1317.7 (354.4) | 1289.7 (365.0) | 1357.5 (335.1) |  | -67.8 (-114.8 to -20.8) | .005 |
| C6 | 1306.2 (358.8) | 1308.9 (376.6) | 1302.5 (333.1) |  | 6.4 (-40.7 to 53.5) | .789 |
| C7 | 1101.2 (394.2) | 1114.0 (400.5) | 1083.3 (384.9) |  | 30.6 (-19.9 to 81.2) | .234 |
| T1 | 626.0 (391.6) | 650.7 (405.7) | 590.6 (368.1) |  | 60.1 (12.1 to 108.0) | .014 |
| Total | 1090.5 (441.4) | 1087.6 (444.1) | 1094.5 (437.7) |  | -6.9 (-29.9 to 16.0) | .554 |
| Longus colli |  |  |  |  |  |  |
| C3 | 292.1 (87.7) | 290.2 (87.1) | 294.6 (88.6) |  | -4.4 (-15.0 to 6.3) | .423 |
| C4 | 267.4 (71.2) | 268.7 (72.5) | 265.8 (69.6) |  | 2.8 (-6.4 to 12.0) | .549 |
| C5 | 262.2 (72.7) | 261.1 (72.3) | 263.8 (73.4) |  | -2.7 (-12.4 to 7.0) | .587 |
| C6 | 267.2 (75.8) | 268.1 (70.0) | 266.0 (83.3) |  | 2.1 (-8.1 to 12.3) | .686 |
| C7 | 276.6 (82.3) | 284.2 (84.6) | 265.9 (77.9) |  | 18.3 (7.8 to 28.8) | .001 |
| T1 | 162.0 (83.1) | 156.6 (86.5) | 169.3 (77.6) |  | -12.6 (-22.8 to -2.5) | .015 |
| Total | 253.6 (90.8) | 253.7 (92.2) | 253.5 (88.8) |  | 0.2 (-4.6 to 4.9) | .950 |

**TABLE S2.** Mean (SD) for unadjusted relative muscle volume for each group (chronic idiopathic neck pain and asymptomatic) calculated per MRI slice for each muscle at each spinal level, with mean difference (95% CI) between groups.

| Relative volume *(mm^3^)* | All | Groups | | | Difference between groups |  |
| --- | --- | --- | --- | --- | --- | --- |
|  | (n=82) | Pain (n=47) | Asymp (n=35) |  | Pain minus Asymp | P |
|  |  |  |  |  |  |  |
| Levator scapulae |  |  |  |  |  |  |
| C3 | 410.1 (194.4) | 379.3 (172.6) | 451.6 (213.8) |  | -72.3 (-96.3 to -48.3) | <.001 |
| C4 | 638.1 (270.2) | 606.5 (237.7) | 679.8 (303.2) |  | -73.3 (-109.1 to -37.5) | <.001 |
| C5 | 875.7 (335.9) | 835.4 (295.7) | 933.1 (379.3) |  | -97.7 (-144.0 to -51.5) | <.001 |
| C6 | 1090.5 (451.0) | 1054.4 (409.0) | 1140.2 (499.5) |  | -85.7 (-146.6 to -24.9) | .006 |
| C7 | 1147.0 (489.7) | 1112.1 (470.8) | 1195.8 (511.5) |  | -83.7 (-147.1 to -20.2) | .010 |
| T1 | 772.1 (377.7) | 735.6 (359.7) | 822.2 (396.0) |  | -86.6 (-132.8 to -40.4) | <.001 |
| Total | 812.3 (446.0) | 779.1 (423.2) | 857.8 (471.9) |  | -78.7 (-102.2 to -55.3) | <.001 |
| Multifidus (with semispinalis cervicis) |  |  |  |  |  |  |
| C3 | 716.8 (146.8) | 738.5 (147.8) | 683.4 (139.4) |  | 55.1 (20.6 to 89.7) | .002 |
| C4 | 925.1 (202.6) | 929.0 (200.1) | 920.0 (206.0) |  | 9.0 (-17.6 to 35.6) | .506 |
| C5 | 1078.4 (270.3) | 1067.6 (257.3) | 1094.0 (287.6) |  | -26.4 (-63.1 to 10.3) | .158 |
| C6 | 1174.0 (318.3) | 1187.8 (314.4) | 1154.9 (322.9) |  | 33.0 (-8.8 to 74.7) | .121 |
| C7 | 1053.8 (301.0) | 1039.5 (288.9) | 1073.8 (316.5) |  | -34.4 (-73.5 to 4.8) | .085 |
| T1 | 851.5 (259.3) | 843.1 (246.6) | 863.1 (275.6) |  | -20.1 (-52.0 to 11.9) | .219 |
| Total | 994.8 (298.2) | 992.7 (290.8) | 997.8 (308.2) |  | -5.1 (-22.0 to 11.7) | .550 |
| Semispinalis capitis |  |  |  |  |  |  |
| C3 | 888.1 (333.2) | 895.8 (338.0) | 877.9 (326.8) |  | 17.9 (-22.7 to 58.4) | .388 |
| C4 | 727.7 (280.9) | 739.4 (290.2) | 712.3 (267.7) |  | 27.1 (-9.2 to 63.3) | .143 |
| C5 | 551.6 (235.2) | 558.7 (241.6) | 541.6 (225.8) |  | 17.1 (-14.2 to 48.4) | .284 |
| C6 | 374.1 (162.9) | 375.8 (158.5) | 371.7 (169.0) |  | 4.1 (-17.5 to 25.7) | .707 |
| C7 | 283.8 (134.9) | 282.6 (130.7) | 285.4 (140.7) |  | -2.9 (-20.4 to 14.6) | .747 |
| T1 | 203.1 (103.6) | 200.4 (104.3) | 206.7 (102.7) |  | -6.3 (-18.8 to 6.3) | .329 |
| Total | 505.4 (335.9) | 507.9 (340.8) | 501.9 (329.1) |  | 5.9 (-11.5 to 23.4) | .504 |
| Splenius capitis (with splenius cervicis) |  |  |  |  |  |  |
| C3 | 849.8 (307.5) | 834.9 (284.9) | 869.8 (334.9) |  | -34.9 (-73.2 to 3.4) | .074 |
| C4 | 752.2 (257.9) | 721.2 (226.4) | 793.2 (289.7) |  | -72.0 (-106.1 to -37.9) | <.001 |
| C5 | 654.4 (220.6) | 631.3 (196.5) | 687.4 (247.6) |  | -56.1 (-86.5 to -25.8) | <.001 |
| C6 | 590.8 (197.0) | 567.3 (169.0) | 623.1 (226.2) |  | -55.8 (-82.6 to -29.0) | <.001 |
| C7 | 497.8 (193.6) | 475.7 (169.4) | 528.8 (219.5) |  | -53.1 (-78.8 to -27.4) | <.001 |
| T1 | 383.2 (159.6) | 368.2 (148.8) | 404.0 (171.5) |  | -35.9 (-55.6 to -16.1) | <.001 |
| Total | 620.4 (278.7) | 598.0 (258.8) | 651.1 (301.2) |  | -53.1 (-67.8 to -38.3) | <.001 |
| Sternocleidomastoid |  |  |  |  |  |  |
| C3 | 868.5 (291.1) | 865.3 (301.9) | 872.7 (276.3) |  | -7.3 (-42.8 to 28.1) | .684 |
| C4 | 1106.0 (330.2) | 1085.3 (326.9) | 1133.3 (333.0) |  | -48.0 (-90.6 to -5.5) | .027 |
| C5 | 1164.1 (323.9) | 1142.0 (323.8) | 1195.8 (321.9) |  | -53.8 (-96.8 to -10.8) | .014 |
| C6 | 1153.7 (332.5) | 1158.5 (336.8) | 1147.1 (326.8) |  | 11.3 (-32.3 to 55.0) | .610 |
| C7 | 947.5 (366.5) | 957.3 (363.1) | 933.7 (371.2) |  | 23.7 (-23.3 to 70.6) | .323 |
| T1 | 509.8 (340.9) | 528.5 (350.9) | 483.0 (324.7) |  | 45.5 (3.7 to 87.2) | .033 |
| Total | 945.2 (404.1) | 942.6 (401.3) | 948.8 (408.1) |  | -6.2 (-27.2 to 14.8) | .564 |
| Longus colli |  |  |  |  |  |  |
| C3 | 251.2 (73.2) | 249.5 (71.2) | 253.4 (75.8) |  | -3.8 (-12.8 to 5.1) | .397 |
| C4 | 226.7 (58.2) | 227.4 (57.3) | 225.9 (59.5) |  | 1.5 (-6.0 to 9.0) | .699 |
| C5 | 217.5 (58.8) | 215.7 (57.3) | 220.2 (60.8) |  | -4.5 (-12.3 to 3.3) | .256 |
| C6 | 218.0 (63.3) | 217.7 (57.7) | 218.4 (70.4) |  | -0.7 (-9.3 to 7.9) | .873 |
| C7 | 226.7 (69.9) | 231.1 (70.0) | 220.5 (69.3) |  | 10.6 (1.7 to 19.5) | .020 |
| T1 | 124.6 (66.3) | 119.7 (68.6) | 131.3 (62.5) |  | -11.7 (-19.8 to -3.6) | .005 |
| Total | 210.0 (77.5) | 209.2 (77.6) | 211.1 (77.5) |  | -1.8 (-5.9 to 2.2) | .370 |

**TABLE S3.** Mean (SD) for unadjusted MFI (%) for each group (chronic idiopathic neck pain and asymptomatic) calculated per MRI slice for each muscle at each spinal level, with mean difference (95% CI) between groups.

| MFI *(%)* | All | Groups | | | Difference between groups |  |
| --- | --- | --- | --- | --- | --- | --- |
|  | (n=82) | Pain (n=47) | Asymp (n=35) |  | Pain minus Asymp | P |
|  |  |  |  |  |  |  |
| Levator scapulae |  |  |  |  |  |  |
| C3 | 12.3 (6.1) | 12.7 (4.5) | 11.8 (7.7) |  | 0.9 (0.1 to 1.7) | .026 |
| C4 | 11.1 (5.7) | 11.1 (3.8) | 11.0 (7.5) |  | 0.1 (-0.7 to 0.9) | .727 |
| C5 | 10.5 (5.5) | 10.4 (3.2) | 10.7 (7.7) |  | -0.3 (-1.1 to 0.5) | .461 |
| C6 | 10.9 (5.6) | 10.7 (3.6) | 11.1 (7.6) |  | -0.4 (-1.2 to 0.4) | .331 |
| C7 | 12.3 (6.0) | 12.3 (4.6) | 12.3 (7.6) |  | -0.03 (-0.9 to 0.8) | .943 |
| T1 | 12.4 (7.0) | 12.4 (4.9) | 12.5 (9.1) |  | -0.1 (-1.1 to 0.8) | .786 |
| Total | 11.6 (6.1) | 11.7 (4.3) | 11.6 (7.9) |  | 0.0 (-0.3 to 0.4) | .799 |
| Multifidus (with semispinalis cervicis) |  |  |  |  |  |  |
| C3 | 35.8 (10.2) | 35.3 (10.1) | 36.6 (10.2) |  | -1.3 (-3.7 to 1.1) | .298 |
| C4 | 24.8 (8.4) | 25.6 (8.9) | 23.8 (7.6) |  | 1.8 (0.7 to 2.8) | .001 |
| C5 | 21.4 (6.6) | 22.1 (6.1) | 20.4 (7.1) |  | 1.7 (0.8 to 2.5) | <.001 |
| C6 | 22.3 (7.1) | 22.8 (6.8) | 21.7 (7.5) |  | 1.1 (0.2 to 2.0) | .021 |
| C7 | 27.0 (8.2) | 28.8 (7.9) | 24.5 (8.0) |  | 4.3 (3.2 to 5.3) | <.001 |
| T1 | 29.7 (8.6) | 31.6 (8.1) | 27.1 (8.6) |  | 4.5 (3.5 to 5.5) | <.001 |
| Total | 25.8 (8.9) | 26.9 (8.8) | 24.3 (8.8) |  | 2.6 (2.1 to 3.0) | <.001 |
| Semispinalis capitis |  |  |  |  |  |  |
| C3 | 18.0 (6.5) | 18.4 (5.4) | 17.6 (7.6) |  | 0.8 (0.0 to 1.7) | .048 |
| C4 | 15.8 (6.8) | 16.0 (5.9) | 15.5 (7.9) |  | 0.5 (-0.3 to 1.4) | .227 |
| C5 | 13.3 (6.0) | 13.5 (4.6) | 12.9 (7.5) |  | 0.7 (-0.2 to 1.6) | .115 |
| C6 | 12.8 (6.2) | 13.1 (5.2) | 12.5 (7.4) |  | 0.6 (-0.2 to 1.5) | .121 |
| C7 | 13.8 (6.8) | 14.2 (5.7) | 13.2 (8.1) |  | 1.1 (0.1 to 2.0) | .025 |
| T1 | 17.3 (8.5) | 17.9 (7.5) | 16.5 (9.6) |  | 1.4 (0.3 to 2.4) | .012 |
| Total | 15.3 (7.2) | 15.6 (6.2) | 14.8 (8.3) |  | 0.8 (0.4 to 1.2) | <.001 |
| Splenius capitis (with splenius cervicis) |  |  |  |  |  |  |
| C3 | 13.3 (6.1) | 13.7 (4.8) | 12.7 (7.5) |  | 1.1 (0.3 to 1.9) | .009 |
| C4 | 12.5 (6.0) | 12.9 (4.7) | 12.0 (7.3) |  | 1.0 (0.2 to 1.7) | .016 |
| C5 | 12.5 (6.2) | 13.0 (5.0) | 11.6 (7.6) |  | 1.4 (0.6 to 2.2) | .001 |
| C6 | 12.4 (6.2) | 12.9 (5.1) | 11.8 (7.5) |  | 1.2 (0.4 to 2.0) | .005 |
| C7 | 12.9 (6.6) | 13.5 (5.8) | 12.1 (7.4) |  | 1.4 (0.6 to 2.3) | .001 |
| T1 | 15.1 (7.6) | 15.8 (7.3) | 14.1 (8.0) |  | 1.8 (0.8 to 2.7) | <.001 |
| Total | 13.2 (6.6) | 13.7 (5.7) | 12.4 (7.6) |  | 1.3 (0.9 to 1.7) | <.001 |
| Sternocleidomastoid |  |  |  |  |  |  |
| C3 | 14.0 (5.9) | 13.9 (4.5) | 14.2 (7.4) |  | -0.3 (-1.1 to 0.5) | .432 |
| C4 | 12.7 (5.5) | 12.6 (4.0) | 12.8 (7.1) |  | -0.2 (-1.0 to 0.5) | .535 |
| C5 | 11.8 (5.5) | 11.4 (3.4) | 12.3 (7.5) |  | -0.9 (-1.7 to -0.1) | .036 |
| C6 | 11.9 (5.7) | 11.5 (3.6) | 12.6 (7.6) |  | -1.1 (-1.9 to -0.2) | .012 |
| C7 | 14.8 (7.3) | 14.5 (5.7) | 15.2 (9.1) |  | -0.7 (-1.7 to 0.3) | .172 |
| T1 | 20.3 (9.5) | 20.2 (8.9) | 20.3 (10.2) |  | -0.1 (-1.3 to 1.1) | .857 |
| Total | 14.4 (7.4) | 14.2 (6.3) | 14.7 (8.7) |  | -0.5 (-0.9 to -0.1) | .018 |
| Longus colli |  |  |  |  |  |  |
| C3 | 13.6 (5.5) | 13.7 (4.1) | 13.6 (7.0) |  | 0.1 (-0.6 to 0.8) | .766 |
| C4 | 14.8 (5.5) | 15.0 (4.1) | 14.7 (7.0) |  | 0.3 (-0.5 to 1.0) | .468 |
| C5 | 16.6 (6.6) | 17.0 (5.9) | 16.1 (7.5) |  | 1.0 (0.1 to 1.8) | .031 |
| C6 | 18.3 (7.0) | 18.7 (6.5) | 17.7 (7.6) |  | 1.0 (0.1 to 1.9) | .036 |
| C7 | 18.0 (7.3) | 18.6 (6.5) | 17.1 (8.2) |  | 1.5 (0.5 to 2.4) | .003 |
| T1 | 23.5 (9.6) | 24.2 (9.0) | 22.5 (10.3) |  | 1.7 (0.5 to 2.9) | .005 |
| Total | 17.5 (7.8) | 17.9 (7.2) | 17.0 (8.6) |  | 0.9 (0.5 to 1.4) | <.001 |
